# Isolation of a recombinant antibody specific for a surface marker of the corneal endothelium by phage display

**DOI:** 10.1038/srep21661

**Published:** 2016-02-23

**Authors:** Simone Dorfmueller, Hwee Ching Tan, Zi Xian Ngoh, Kai Yee Toh, Gary Peh, Heng-Pei Ang, Xin-Yi Seah, Angela Chin, Andre Choo, Jodhbir S. Mehta, William Sun

**Affiliations:** 1 Experimental Therapeutics Centre, 31 Biopolis Way, Nanos level 3, Singapore, 138669 Singapore; 2 Tissue Engineering and Stem Cell Group, Singapore Eye Research Institute, Singapore; 3 Bioprocessing Technology Institute, 20 Biopolis Way, Centros Level 4, Singapore, 138668 Singapore; 4 Department of Biomedical Engineering, Faculty of Engineering, National University of Singapore, Singapore; 5 Institute of Bioengineering and Nanotechnology, Synthetic Biosystems, 31 Biopolis Way, #04-01 Nanos, Singapore, 138669 Singapore; 6 Department of Physiology, Yong Loo Lin School of Medicine, National University of Singapore, Singapore

## Abstract

Cell surface antigens are important targets for monoclonal antibodies, but they are often difficult to work with due to their association with the cell membrane. Phage display is a versatile technique that can be applied to generate binders against difficult targets. Here we used antibody phage display to isolate a binder for a rare and specialized cell, the human corneal endothelial cell. The human corneal endothelium is a medically important cell layer; defects in this layer account for about half of all corneal transplants. Despite its importance, no specific antigens have been found to mark this cell type. By panning a phage library directly on human corneal endothelial cells, we isolated an antibody that bound to these cells and not the other types of corneal cells. Subsequently, we identified the antibody’s putative target to be CD166 by immunoprecipitation and mass spectrometry. This approach can be used to isolate antibodies against other poorly-characterized cell types, such as stem cells or cancer cells, without any prior knowledge of their discriminating markers.

The corneal endothelium is a monolayer of metabolically active cells that lines the inner surface of the cornea. It has the important function of regulating fluid flow into the corneal stroma, thereby maintaining its clarity^1^,^2^. Because human corneal endothelial cells (hCECs) do not regenerate *in vivo*^3^,^4^, excessive loss of these cells due to aging, disease, or surgical trauma^5^ will eventually lead to corneal swelling and visual impairment^6^. Endothelial dysfunction is a leading cause of corneal blindness worldwide and the major indication for corneal transplantation^7^. While advanced surgical procedures can selectively replace the endothelial layer (e.g. Descemet’s stripping endothelial keratoplasty or DSEK), the number of patients requiring transplants far exceeds the number of available donor corneas^8^. To overcome the shortage of donor tissue, current research aims to induce proliferation of primary hCECs in culture^9^,^10^,^11^,^12^, or to generate hCECs from progenitor or stem cells^13^,^14^,^15^. Progress in this area is hampered by a lack of definitive markers to identify hCECs.

Two widely used markers reported for cultured hCECs are pump-associated protein Na^+^/K^+^-ATPase^16^ and tight-junction protein ZO-1^17^. In the cornea, the co-expression of these proteins indicates the presence of functional components on the corneal endothelium, but these markers are nonspecific and are expressed in other tissues including the heart^18^, brain^19^, and kidney^20^. Other groups have previously reported the generation of antibodies against corneal endothelial cells. Several groups used a whole-cell immunization approach to isolate mouse monoclonal antibodies against hCECs^21^,^22^,^23^,^24^, but these antibodies cross-reacted with several other human tissues.

Since hCEC-specific cell surface antigens are lacking, the goal of this study was to identify antibodies that can bind to the surface of corneal endothelium and cultivated hCECs, but do not bind stromal keratocytes and fibroblasts. Several approaches could be used to identify antibodies against cell surface markers. If the surface antigen is known, then one strategy would be to express the soluble extracellular domain, which could be used to immunize animals or be used as target for antibody phage display. Other methods include whole-cell immunization or panning on whole cells using phage display. Since no specific markers have been identified for hCEC, we opted to use antibody phage display to isolate binders for these cells. Antibody phage display is a versatile technique that can be used against a wide variety of targets, including cell surface antigens^25^,^26^,^27^. In this study, we used a phage library based on the naïve human repertoire to isolate a single-chain variable fragment (scFv) that binds selectively to the human corneal endothelium. We reformatted it to a full immunoglobulin and identified its target antigen by immunoprecipitation and mass spectrometry.

## Results

### hCEC specific enrichment of the ETC-H1 human scFv library

To find antibodies that will recognize hCECs, we used the in-house generated ETC-H1 library, a fully human scFv library displayed on the pIII protein of KM13 helper phage. The first round of bio-panning was performed on intact human corneal tissue to ensure a native environment of endothelial cells. The enriched library, named ETC-H1-C1, was further panned on either intact corneas or on cultured corneal endothelial cells. For selection on cultured hCECs, cells were grown as a monolayer on glass slides and then mounted into microfluidic chambers^25^. The enriched libraries from the different rounds were named according to the method of panning. For example, ETC-H1-C1M2 would denote a sub-library that was generated from one round of panning on intact corneal tissue and two rounds of panning on cultured hCECs.

As shown previously by other groups, hCEC cultured *in vitro* for extended passages tend to exhibit a fibroblastic morphology^28^. We therefore hypothesized that hCECs and corneal stromal fibroblasts would share a significant portion of their surface epitopes. To preferentially select for phages that bind hCEC-specific epitopes, we pre-absorbed the ETC-H1 library with 10^8^ stromal fibroblasts. This negative selection step was also performed after each round of panning on the intact corneas. For panning on cultured hCECs, we devised a subtraction scheme where the phage library was circulated through five micro-chambers of stromal fibroblasts before entering the chamber containing hCECs. Microscopic examination of the culture slide after panning showed that the fibroblast and hCEC monolayers remained intact throughout the procedure (data not shown).

**Figure 1:**
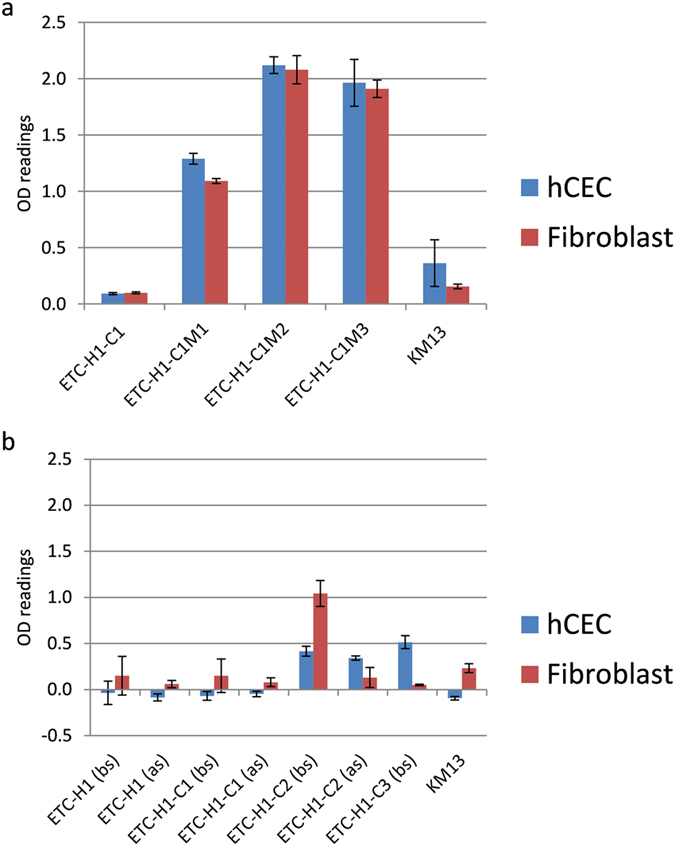
Enrichment of the ETC-H1 phage library with different panning rounds as determined by polyclonal phage ELISA. Phages were tested on hCECs (blue bars) or fibroblasts (red bars) seeded in 96-well plates, and binding was detected by M13-specific antibody conjugated to horseradish peroxidase. The helper phage KM13 was used as a negative control. (**a**) Enrichment of ETC-H1 after one round of panning on corneal tissue and 3 rounds on hCECs in microfluidic chambers. 3 × 10^10^ phages were tested per well. There was no significant difference in the ELISA signals between hCEC and fibroblasts for all libraries (ANOVA). (**b**) Enrichment of the ETC-H1 library with increasing rounds of panning on intact human corneas. ELISAs were performed before (bs) and after (as) subtraction of the libraries with excess fibroblasts. 9.5 × 10^8^ phages were tested per well. There was significant difference in OD readings between hCEC and fibroblasts for ETC-H1-C2 (as) (two-tailed Student T-test, P = 0.03) and ETC-H1-C3 (bs) (P = 0.005). Error bars indicate standard deviations (N = 3).

We monitored the enrichment of the phage library after each panning round by performing polyclonal phage ELISA on cultured hCECs or fibroblasts. For the libraries selected on cultured hCECs in microfluidic chambers (Fig. 1a), the increase in OD readings over several selection rounds indicated progressive enrichment for hCEC-binding phages (blue bars). However, the enrichment was not specific for hCECs since there was a comparable increase in the ELISA signal for fibroblasts (red bars). Co-enrichment of fibroblast-specific phage particles while panning on hCECs supported the idea that both cell types share a significant number of surface epitopes.

The results from panning on intact corneas showed that continuous subtraction with fibroblasts after each round was necessary to obtain a hCEC-specific library. As shown in Fig. 1b, only after two rounds of subtraction with excess fibroblasts could the polyclonal ELISA generate an hCEC-specific signal. Hence, this approach of negative selection was more effective than that used for the microfluidic chambers. But while specificity increased during panning with corneal tissue, affinity seemed to be compromised as the ELISA signal for the ETC-H1-C3 library (OD ≈ 0.5) was much lower than that for the ETC-H1-C1M3 library (OD ≈ 2.0). Possible reasons include that changes in surface antigen composition occurred after the hCECs were cultured *in vitro*, or that extensive subtraction of the library with fibroblasts removed the high affinity binders to epitopes shared by both cell types.

### Selection of a scFv specific for cultured hCECs

We chose to screen the enriched library ETC-H1-C3, which was panned over three rounds on human corneas, for individual binders to cultured hCEC by monoclonal soluble scFv ELISA. Based on the results from the polyclonal phage ELISA we expected to find binders which were specific for hCEC only, but possibly with low affinity. We also screened clones from the ETC-H1-C1M2 and ETC-H1-C1M3 libraries, which yielded higher OD readings on the polyclonal ELISA, to see if we could isolate hCEC-specific binders with higher affinity.

**Figure 2:**
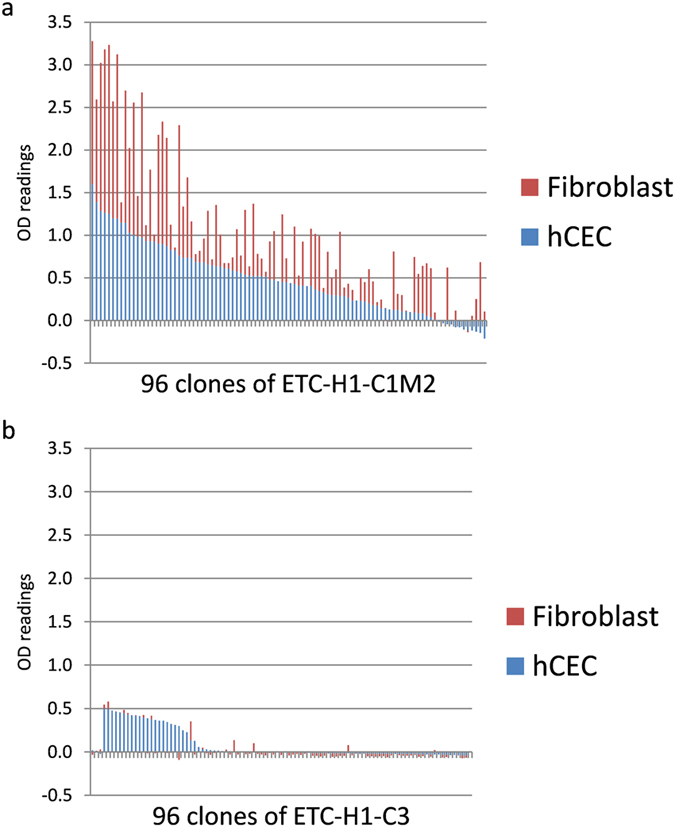
Screening of individual clones from enriched libraries ETC-H1-C1M2 (**a**) and ETC-H1-C3 (**b**) by monoclonal soluble scFv ELISA. hCECs and stromal fibroblasts were seeded in 96 well plates and incubated with soluble scFv. Specific binding was detected by c-myc antibody conjugated to horseradish peroxidase. OD readings of the different clones generated on hCECs were sorted from highest to lowest and plotted with the corresponding signals on fibroblasts.

Figure 2a represents a typical monoclonal soluble scFv ELISA on hCEC and fibroblast for the ETC-H1-C1M2 library, whereas Fig. 2b shows a typical result for the ETC-H1-C3 library. For both graphs, the OD readings from hCECs (blue bars) were sorted from highest to lowest and plotted with the respective signals on fibroblasts (red bars). The highest OD readings were obtained for clones of the ETC-H1-C1M2 library which were specific for both cell types, i.e. they recognized an antigen shared by both hCECs and fibroblasts (Fig. 2a). Clones that were specific for hCEC showed comparatively lower affinity, regardless of which library was screened (Fig. 2b). This reflected the previously observed difference in polyclonal phage ELISA, as the high signals from phages that bound both hCECs and fibroblasts would mask the lower readings generated by the hCEC-specific clones.

A total of 960 individual clones from ETC-H1-C3, 192 clones from ETC-H1-C1M2, and 96 clones from ETC-H1-C1M3 were tested in parallel as soluble scFv on cultured hCECs and fibroblasts. Of the 960 ETC-H1-C3 clones screened, 65 showed specific binding to hCECs (6.8%). For ETC-H1-C1M2 and ETC-H1-C1M3 libraries, we identified 8 (4.2%) and 6 (6.3%) putative positives, respectively. Sequence analysis grouped these 79 clones into 20 individual non-redundant sequences, whereby a clone named C3-1-A10 was represented 48 times. Two other clones, C3-2-D12 and C1M2-2-B8, each appeared 7 times, and the remaining 17 sequences were found only once or twice. The amino acid sequences of the CDR’s for the 20 clones are shown in Supplementary Tables S1 and S2.

**Figure 3:**
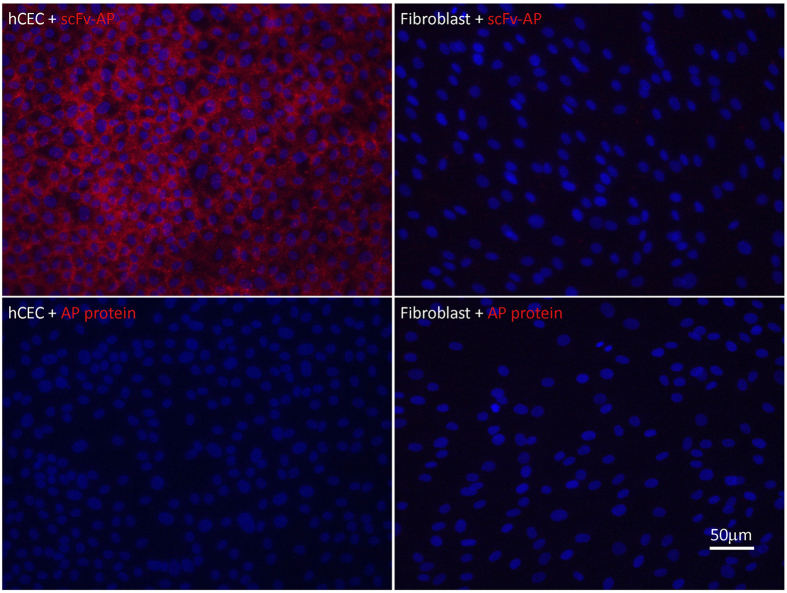
Immunofluorescence microscopy on hCECs and stromal fibroblasts. Cell cultures of hCECs (95 K/well) and stromal fibroblasts (300 k/well) were seeded onto a 12-well multi slide, fixed with formaldehyde and stained with C1M2-2-B8 scFv-AP fusion (100 μg/ml) or AP protein without scFv (100 μg/ml). Specific binding was detected by Vector Red alkaline phosphatase substrate. Nuclei of cells were stained by DAPI.

**Figure 4:**
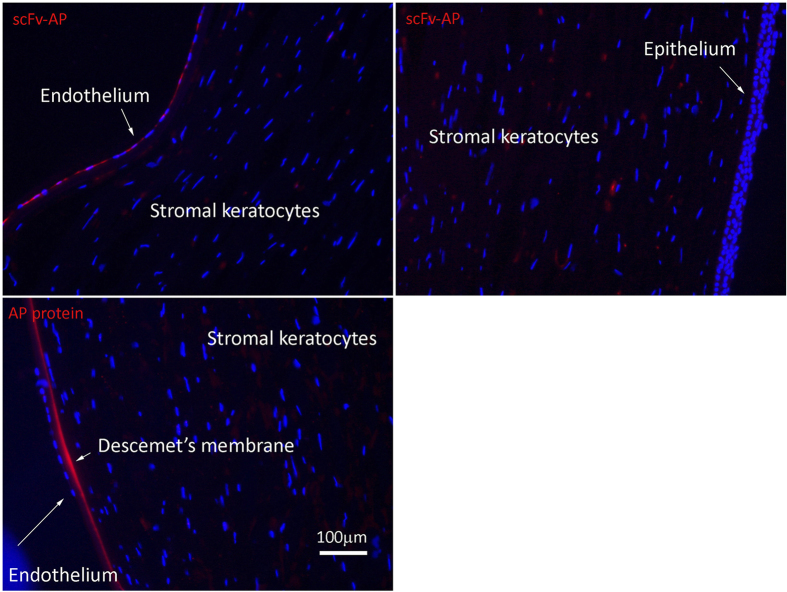
Immunofluorescence microscopy on human cornea tissue sections stained with C1M2-2-B8 scFv-AP fusion. Formaldehyde-fixed cornea sections were incubated with C1M2-2-B8 AP-fusion protein (20 μg/ml) or AP protein (20 μg/ml) without scFv. Specific binding was detected by Vector Red Alkaline phosphatase substrate. Nuclei of cells were stained by DAPI.

All 20 scFv’s were subcloned into the pET21d-AP expression vector for large scale expression as a fusion protein to the bacterial alkaline phosphatase^29^. The obtained yields for the 75 kD scFv-AP fusion protein varied between 0.4 mg and 0.0015 mg per litre of bacterial culture for the different clones (see Supplementary Table S3). Specificity of the scFv-AP constructs was tested by immunohistochemical staining on cultured hCECs and fibroblasts at a range of 20 μg/ml to 500 μg/ml scFv-AP protein. The C3-1-A10 and C3-2-D12 scFv-AP fusion proteins reacted with fibroblasts at elevated concentrations, whereas the C1M2-2-B8 scFv-AP construct detected cultured hCECs at a low concentration of 20 μg/ml and was negative on fibroblasts at 500 μg/ml (Fig. 3; see Supplementary Fig. S1 for phase contrast images). Clones that did not show a bright signal on hCEC or showed cross reactivity to fibroblasts were excluded. An example of a clone that bound both cell types was C1M2-2-B2 (see Supplementary Fig. S2). Tissue specificity for C1M2-2-B8 scFv-AP was further tested on frozen human cornea sections (Fig. 4). The scFv reacted intensely with the corneal endothelial monolayer, but did not stain the stroma or the epithelium.

### Immunohistochemistry analysis of reformatted IgG-B8

Because C1M2-2-B8 scFv-AP showed the desired immunostaining profile, we reformatted it into a full immunoglobulin (IgG-B8) for further downstream characterization. The variable heavy domain of the V3/J4/D3 subtype was fused to a human IgG1 framework and the variable light chain domain of the V6/LJ3 subtype to a human kappa constant region. The resulting IgG-B8 clone was transiently expressed in HEK293F cells and the expressed immunoglobulin was purified by protein G affinity chromatography with a yield of 1.3 mg per 250 ml of transfected culture.

**Figure 5:**
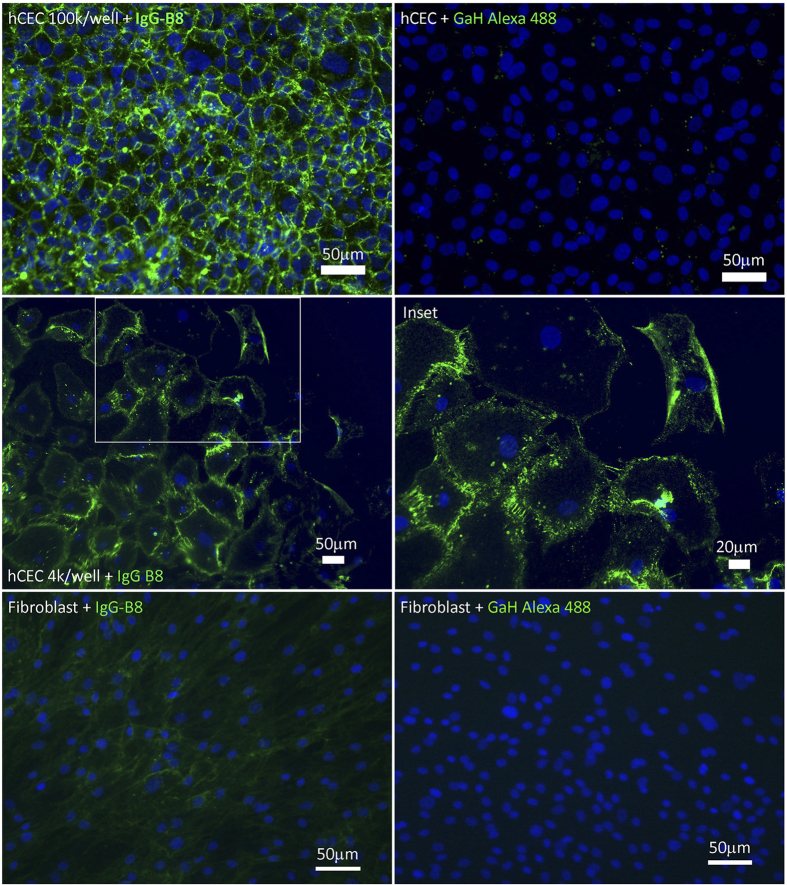
Immunofluorescence microscopy of full-length IgG-B8 on hCECs and stromal fibroblasts. Cell cultures of hCECs (100 K/well, 4 K/well) and stromal fibroblasts (300 k/well) were fixed with formaldehyde and stained with IgG-B8 (25 μg/ml). Specific binding was detected by goat anti-human antibody (GaH) conjugated to Alexafluor 488. Nuclei of cells were stained by DAPI.

Binding specificity of the IgG-B8 was tested on cultured hCECs and stromal fibroblasts as well as on human cornea tissue sections. As observed for the scFv-AP version of the antibody, the IgG-B8 stained intensely the cell membrane of confluent hCECs (Fig. 5). Due to the increased avidity of the two antigen binding sites of an IgG in contrast to a monovalent scFv, we could lower the IgG-B8 concentration to 2 μg/ml for immunocytochemistry. In sub-confluent conditions, where hCECs showed less cell-to-cell contact, we observed brightly stained membrane strings between adjoining cells. This could indicate that the B8 antigen was involved in mediating cell-cell contact, which is an important morphological feature of hCECs. Surprisingly, we saw weak cross-staining of the IgG-B8 on cultured stromal fibroblasts. The IgG-B8 antigen might be expressed at low levels in stromal fibroblasts, and its detection was possible due to the increased avidity of the full IgG molecule.

**Figure 6:**
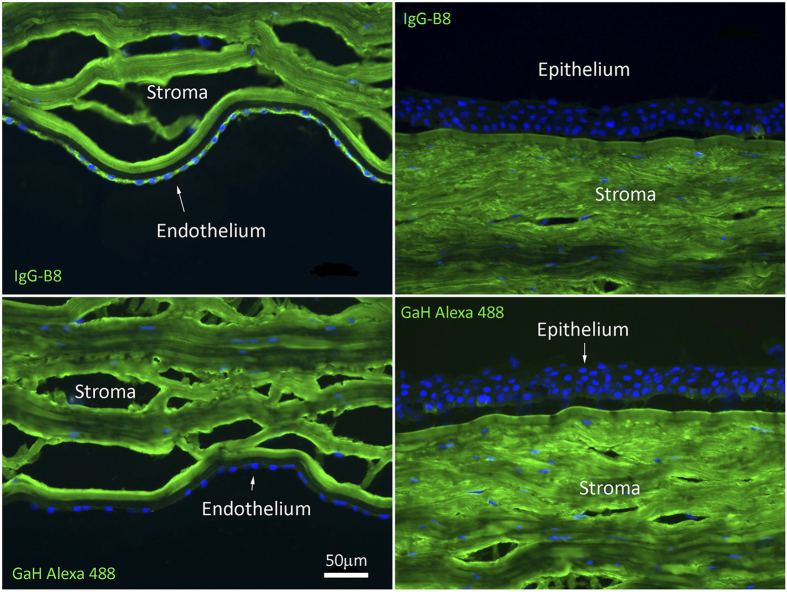
Immunofluorescence microscopy of human cornea tissue sections stained with IgG-B8 antibody. Formaldehyde-fixed human cornea sections were incubated with IgG-B8 (25 μg/ml) and specific binding was detected by goat anti-human antibody (GaH) conjugated to Alexafluor 488. Nuclei of cells were stained by DAPI.

Similar to the results obtained for the C1M2-2-B8 scFv-AP construct, IgG-B8 stained brightly the endothelial layer in the human corneal tissue sections (Fig. 6). Due to the strong reaction of the goat anti-human secondary antibody against the corneal stroma, we could not conclusively assess IgG-B8 staining of this layer. There was no specific staining of the corneal epithelium. Overall, our results showed that the IgG-B8 target antigen was localized to the endothelial layer and not the other layers of the human cornea.

### Flow cytometry analysis of antibody labelled hCECs and stromal fibroblasts

**Figure 7:**
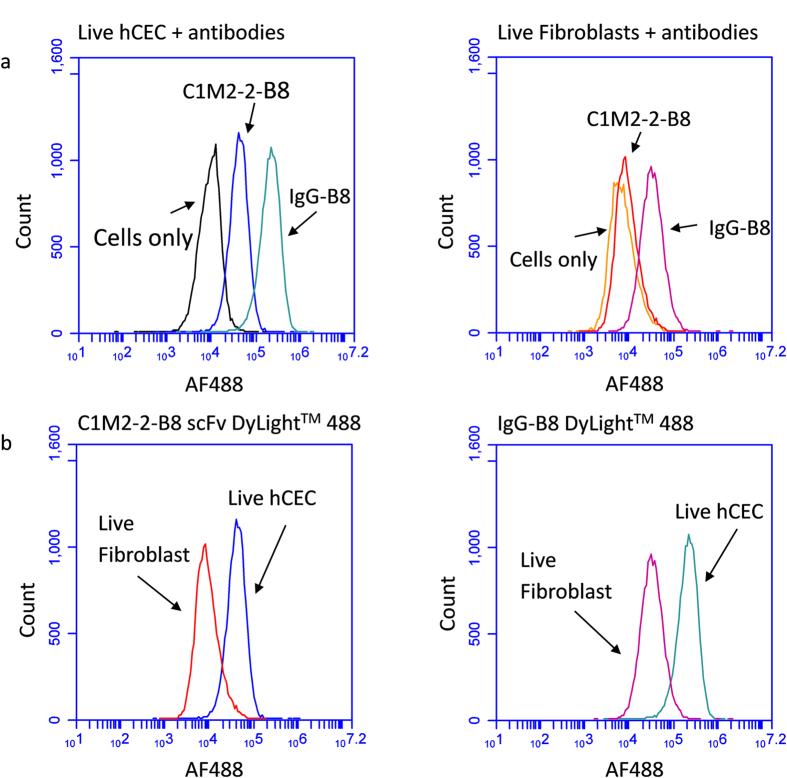
Flow cytometry analysis of antibody-labelled hCECs and stromal fibroblasts. hCECs or stromal fibroblasts were incubated with C1M2-2-B8 scFv or IgG-B8 full-size antibody conjugated to DyLight™ 488 (4 μg/ml). (**a**) The histogram for unlabelled hCECs (black) is overlaid with those for scFv and IgG-B8 labelled cells as indicated. Similarly, the histogram for unlabelled stromal fibroblasts (orange) is overlaid with those for scFv and IgG-B8 labelled fibroblasts. (**b**) The histograms for C1M2-2-B8 scFv labelled hCECs and stromal fibroblasts were shown on the same graph, as for the histograms for IgG-B8 labelled hCECs and stromal fibroblasts. The peak separation between fibroblasts and hCECs were similar in magnitude with either scFv or IgG labelling.

A potential use for hCEC-specific antibodies is the separation of endothelial cells by fluorescent-activated cell sorting from contaminating fibroblasts. Therefore, we characterized the C1M2-2-B8 scFv and its reformatted IgG-B8 by flow cytometry. For this purpose we expressed the C1M2-2-B8 without the AP fusion and conjugated the purified scFv as well as the IgG-B8 to DyLight™ 488. Cultured hCECs and stromal fibroblasts were labelled with the scFv and IgG versions of B8 and analysed by flow cytometry. Consistent with the immunostaining results, both antibody formats caused a shift in the histogram of labelled hCECs when compared to unlabelled cells, with the IgG-B8 showing a greater signal over the scFv. A very small shift was seen when stromal fibroblast were stained with C1M2-2-B2 scFv, but the IgG-B8 showed a significant reaction (Fig. 7a). This result was consistent with that of the immunocytochemistry on cultured stromal fibroblasts where no signal was observed for the C1M2-2-B8 scFv AP construct, but a weak signal was present for the IgG-B8.

When the histograms for hCEC and fibroblast cells stained with C1M2-2-B8 scFv were shown in the same plot, the peak separation was similar in extent to that for the histograms of the IgG-B8 labelled cells (Fig. 7b). Although the IgG-B8 yielded a brighter signal when compared to the scFv, the ability of the two antibody formats to discriminate between hCECs and the stromal fibroblasts was identical. The flow cytometry experiments were also performed on fixed endothelial cells and stromal fibroblast and no significant difference between live or formalin fixed cells was observed (data not shown).

### Identification of the IgG B8 target antigen

**Figure 8:**
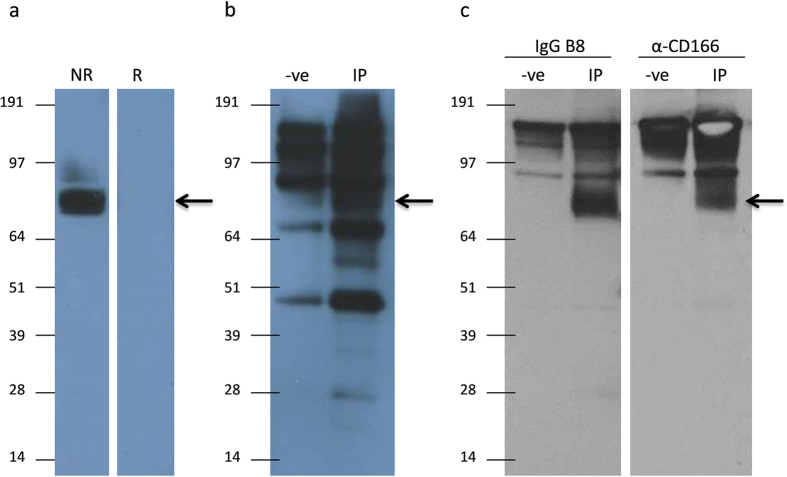
Identification of IgG-B8 antigen target. (**a**) hCEC total lysate probed with IgG-B8 under non-reducing (NR) and reducing (R) conditions. (**b**) IP on hCEC total cell lysate using the IgG-B8 enriched a protein (arrow) which was excised and analysed by LC/MS-MS. This band was not enriched when IgG-B8 was omitted in the experiment (−ve). (**c**) IgG-B8 IP samples were probed with IgG-B8 and a commercial anti-CD166 antibody.

To evaluate the molecular size of the IgG-B8 target antigen, hCEC total lysate was separated under reducing and non-reducing conditions and probed with IgG B8 by western blotting. As shown in Fig. 8a, IgG-B8 bound to a ~80 kD band only under non-reducing conditions, suggesting that the antibody recognized a conformational epitope. Immunoprecipitation of hCEC lysates with IgG-B8 successfully enriched a ~80 kD band (Fig. 8b) and the band was excised from a silver stained gel and subjected to LC/MS-MS analysis for antigen identification. A database search of MS data showed a 47% peptide sequence match with ALCAM/CD166. The target was validated by using a commercial anti-CD166 antibody (Abcam cat# ab109215) to probe the IgG-B8 IP samples. The IgG-B8 and commercial CD166 antibodies detected protein bands of similar size (Fig. 8c).

## Discussion

Surface markers that uniquely identify a particular cell type are valuable targets for antibody discovery, either to be used as diagnostic biomarkers or as therapeutic targets. Because there is great interest in generating hCECs for transplantation, either from expanding existing donor pools^10^,^11^,^30^ or from differentiating stem cells^13^,^14^,^15^, antibodies that can specifically bind this cell type would be very useful.

Previous attempts to find hCEC-specific antibodies included whole cell immunization of mice^21^,^22^,^24^ or analysis of hCEC gene expression to identify discriminating markers^31^,^32^,^33^. This study demonstrated the use of antibody phage display as an additional approach to generate hCEC-specific antibodies. Our results showed that although it was quite difficult to isolate an hCEC-specific antibody due to the similarity of surface antigens between hCECs and stromal fibroblasts, we were able to overcome this hurdle through a customized selection and subtraction scheme. We began our library selection directly on the intact human cornea to preclude any changes to the hCECs due to *in vitro* culture. In addition, we subjected the library to extensive subtraction with stromal fibroblasts after each round. Such a selection and subtraction scheme would not be possible through the classical approach of animal immunization and thus demonstrates the power of phage display technology.

In addition to panning of our phage library on intact human corneas, we explored the method of panning with cultured hCECs grown as a monolayer in a microfluidic chamber^25^. Panning with the microfluidic chamber allowed us to reduce the number of cells required when compared to panning with cells in suspension. We placed five chambers of stromal fibroblasts in series with one chamber of hCECs to enable simultaneous selection and subtraction of target phages. As shown by polyclonal ELISA, however, this subtraction scheme was not as effective as that performed with the fibroblasts in suspension during panning with the intact corneas. The polyclonal ELISA also indicated that stringent negative selection could increase library specificity but at a cost of decreasing affinity. Nevertheless we were able to identify the scFv clone B8 from the ETC-H1-C1M2 library, suggesting that rare hCEC-specific clones were hidden among the majority that bound both cell types.

The C1M2-2-B8 scFv showed selective binding to the corneal endothelial layer and not the other layers of the human cornea. When reformatted into a full human IgG, the IgG-B8 retained its binding to the hCEC, but also showed slight cross-reactivity to the stromal fibroblasts. The immunohistochemistry results were consistent with those obtained by flow cytometry. The cross-reactivity to fibroblasts could be due to the increased avidity of the IgG-B8 and the presence of low amounts of the target antigen on the stromal fibroblasts. We subsequently identified the IgG-B8 target antigen as ALCAM or CD166. Interestingly, Ding *et al.*^24^ independently isolated a mouse monoclonal antibody against the same antigen using an entirely different approach of whole-cell immunization. FACS screening of a commercial panel of antibodies against human cell surface markers also identified CD166 as a marker for cultured hCECs that maintained a non-fibroblast phenotype^34^. The exact biological role of CD166 is unclear, but it has been shown to be involved in cell adhesion, growth and migration^35^. The expression of CD166 apparently decreased when hCECs began to adopt a fibroblastic morphology in culture. This observation is in line with our result where IgG-B8 immunostaining yielded a strong signal on hCECs but a weak signal on stromal fibroblasts.

While the B8 antibody showed preferential binding to hCECs and not the other corneal layers, it is not entirely specific for this cell type. Ding *et al.* has shown that some antibodies to CD166 cross reacted with other cell types including embryonic stem cells and lung fibroblasts^24^. Therefore, our B8 antibody would not be able to distinguish hCEC from other cells that also express CD166. Future work would include further characterization of the B8, such as epitope mapping, to confirm the target antigen. In addition, efforts could be put into screening other phage libraries and subtraction with more cell types during panning, including those that already express the CD166 antigen.

In conclusion we showed that phage display is a powerful technology to isolate antibodies with affinity for cell surface markers. Biopanning strategies can be customized to address specific experimental hurdles and *in vitro* selection offers the possibility to monitor and fine tune the enrichment process. We isolated an antibody from our phage library that bound hCECs and not stromal fibroblasts, as shown by immunohistochemistry and flow cytometry. We then identified the target antigen to be CD166 by immunoprecipitation and mass spectrometry. The same approach can be used to discover antibodies and discriminating markers for other rare and uncharacterized cell types, such as stem cells or cancer cells.

## Materials and Methods

### Antibody phage display library construction and preparation

The ETC-H1 library was constructed based on the naïve human repertoire in the scFv format. Human spleen, peripheral blood, and lymph node RNAs were obtained from commercial sources (see Supplementary Table S4). The RNAs were reverse transcribed using M-MuLV reverse transcriptase. VH and VL fragments were amplified using degenerate primers^36^ and cloned into the pIT2 vector^37^ using a two-step process. The library was electroporated into TG1 competent cells and 10^10^ independent clones were plated out on large square dishes. DNA sequencing of fifty random clones showed that they all had different sequences. The colonies were harvested, pooled, and stored in aliquots at −80 °C. 100 μl of the phage library in TG1 were inoculated into 50 ml of 2 × TY medium, 100 μg/ml ampicillin, 1% glucose. 10 ml of the culture were mixed with 10^11^ hyperphage^38^ or KM13 helper phage^37^. The rescue of the phages was carried out essentially as described in Marks *et al.*^39^. The phages were titrated by phage ELISA (Hyperphage) or by TG1 infection (KM13 helper phage) and stored at 4 °C or used directly for panning or subtraction against fibroblasts.

### Subtraction of fibroblast specific phages

Human stromal fibroblast were isolated and cultivated as described by Cheong *et al.*^31^. Approximately 10^7^–10^8^ Fibroblast cells were grown to nearly 90% confluence and disassociated with enzyme free PBS based disassociation buffer (Gibco). Cells were resuspended in the prepared phage library, incubated for 2 h at 4 °C and fibroblasts with bound phages removed. Subtraction was repeated twice and remaining unbound phages were titered by phage ELISA (Hyperphage) or TG1 infection (KM13).

### Phage panning on human corneas

Human donor corneas were procured from Lions Eye Institute for Transplant and Research Inc. (Tampa, FL, USA), with written informed consent from the next of kin and adherence to the principles outlined in the Declaration of Helsinki. Research corneas deemed unsuitable for transplantation, each with an endothelial cell count of at least 2,000 cells per mm^2^, were obtained for this study. Corneoscleral tissues were preserved in Optisol-GS (Bausch & Lomb, Rochester, NY, USA) at 4 °C until they were processed within 14 days of preservation. The donor corneas were washed with 50% v/v Endothelial-SFM (Life Technologies) /50% PBS and subsequently blocked for 2 h with gentle agitation at RT in Optisol containing 3% BSA or 2% skimmed milk on alternate panning rounds. After blocking each cornea was washed with cold SFM/PBS, submerged in 4 ml of SFM/PBS, 1% BSA and 10^12^–10^13^ fibroblast subtracted phages and incubated for 2 h at 4 °C with gentle agitation. The phages were aspirated off and the cornea was washed extensively with cold SFM/PBS to remove unbound phages. The DM-endothelial layer was peeled off, and bound phages were eluted in 5 mM Trypsin 1 mM CaCl_2_ in PBS and mixed with log-phase TG1. Infected TG1 were plated out and colonies were scraped and stored at −80 °C.

### Phage panning on cell monolayers in microfluidic chambers

The microfluidic chambers were designed as described^25^ with some modifications and manufactured by CSEM (Switzerland). Cultivation of hCECs was carried out as previously described^30^,^31^. Primary hCECs and corneal stromal fibroblast cells were seeded into two wells of chamber slides (NUNC Lab-Tek) coated with FnC and cultivated to nearly 90% confluence. The culture slide chamber walls were removed and the cell-coated slide was assembled into the micro-chamber device. Cold blocking, phage and wash buffers were pumped through the device by a peristaltic pump with silicon tubing at a flow rate of 0.3 ml/min at 4 °C. For the blocking and panning steps, the hCEC loaded micro-chamber was connected last in line behind five fibroblast-loaded micro-chambers. Blocking buffer (3% BSA and 2% skimmed milk on alternate panning rounds) was circulated for 2 h through the system, followed by 10^12^–10^13^ phages in 3 ml PBS for 3 h. The fibroblast-loaded micro-chambers were removed and PBS was pumped through the hCEC-loaded micro-chamber for 2 h. The device was disassembled and the hCEC-specific phages were eluted by spotting 100 μl to 150 μl 5 mM Trypsin, 1 mM CaCl_2_ in PBS on top of the monolayer. The eluted phages were aspired off and mixed with log-phase TG1. Infected TG1 were plated out and colonies were scraped and stored at −80 °C.

### Cell based ELISA

To monitor efficient subtraction of fibroblast-specific and enrichment of hCEC-specific phages, polyclonal phage libraries before and after subtraction and/or panning round were collected and tested on fibroblasts and hCECs. Cells were grown to 90% confluency in FnC-coated 96 well plates, fixed with 10% formaldehyde and blocked with 400 μl 3% BSA per well. Hyperphage or KM13 helper phage were included as negative control. Bound phages were detected by HRP-conjugated anti-M13 antibody (GE Healthcare) and the assay was developed by addition of TMB (Promega). Reaction was stopped by the addition of H_2_SO_4_ and reading taken at OD_450nm_.

To screen for hCEC-specific clones, soluble monoclonal scFv was expressed directly from the pIT2 vector. Well-isolated colonies were inoculated o/n at 37 °C in 2 × TY, ampicillin, 1% glucose. A vector only clone was used as negative control. The overnight cultures were mixed 1:6 with 2 × TY, ampicillin, 1% glucose and incubated for 4–5 h at 37 °C. Medium was exchanged with 2 × TY, ampicillin, 0.4 M sucrose, 1 mM IPTG, and cultures were incubated o/n at 30 °C and pelleted. 100 μl of the supernatant was tested in parallel on cultivated hCEC and fibroblast as described for the phage ELISA, except that the detection was done by a HRP-conjugated c-myc specific antibody (Pierce).

### Recloning, protein expression and purification of scFv

The scFv was PCR amplified from the pIT2 vector as a pelB-scFv construct and subcloned into a pet21d bacterial expression vector with or without integrated bacterial alkaline phosphatase^29^ upstream of a C-terminal hexa-histidine tag by the *Nhe*I and *Not*I restriction sites. Protein expression was driven by the T7 promoter with IPTG inducible lac operon in BL21 *E. coli* host cells. The 2YT, ampicillin, 2% glucose o/n culture of a well isolated colony was inoculated 1:100 in 1 L 2 × TY, ampicillin, 0.1% glucose expression culture and incubated at 37 °C until OD_600nm_ of 0.6–0.8. Protein expression was induced by 0.1 mM IPTG for 4–5 h at 25 °C, and pelleted cells were incubated for 2 h on ice in 50 ml 1 × TES buffer (0.2 M Tris-HCl pH 8, 0.5 mM EDTA, 0.5 M sucrose) supplemented with Complete Protease Inhibitor Cocktail without EDTA (Roche). Cells were pelleted and the supernatant was filtered (0.2 μm) and dialyzed twice against PBS using a 10 kDA cut-off snake skin dialyzing tube. The scFv or scFv-AP fusion protein was purified by Ni-NTA affinity chromatography (Qiagen) and anion exchange (Mono Q, GE healthcare). The purified proteins were quantified by spectroscopy using a Nanodrop 1000.

### Reformatting to full-size Ab, protein expression and purification

The variable light domain was amplified using primers which introduced an *Apa*LI and *Pst*I restriction site for cloning into a mammalian expression vector with integrated human kappa constant region^40^. The variable heavy chain domain was amplified using primers which introduced a *Mfe*I and XhoI restriction site for cloning the VH in front of a human IgG1 CH1, CH2 and CH3 region of the same expression vector. The full-size human IgG1 construct was transfected into the FreeStyle™ MAX 293F system according to manufacturer’s (Invitrogen) protocol for transient expression. Seven days post transfection, the full-size human IgG1 was purified from the culture medium by protein G (Pierce) affinity chromatography according to manufacturer’s protocol.

### Immunohistochemistry

For immunohistochemistry of frozen sections, human donor cornea was rinsed in PBS twice and immersed in OCT medium and frozen at −80 °C until sectioning. Eight-micron thick sections were cut using a MicromHM550 cryostat and collected on glass slides and air-dried before storage at −80 °C. For *in vitro* staining of primary hCECs and corneal stromal fibroblasts, cells were seeded on multi-test slides (MP Biomedicals), pre-coated with FnC. Cells or tissue sections were fixed in 10% formaldehyde, blocked with goat serum (Millipore) and incubated with primary antibodies (scFv AP or IgG). Bound scFv-AP was detected by the Vector Red alkaline phosphatase substrate kit (Vector Labs), and bound IgG by a goat anti-human Alexa488 conjugated secondary antibody. Cells were counter-stained with DAPI. Slides were viewed under a fluorescence microscope.

### Flow cytometry

Primary hCECs and fibroblasts were dissociated as described earlier, blocked with goat serum and incubated for 45 min at 4 °C with scFv or IgG conjugated to DyLight™ 488 (Innova Bioscience). Conjuation with DyLight 488 was performed according to the manufacturer’s protocol. The cells were resuspended in PBS and analyzed by flow cytometry (Accuri C6, BD).

### Immunoprecipitation and mass spectrometry

Immunoprecipitation with full-size IgG B8 on total cell lysate of hCECs and subsequent antigen target identification using mass spectrometry (LC/MS-MS) were performed as described by Ding *et al.*^24^. More detailed methods are provided in the Supplementary Information.

## Additional Information

**How to cite this article**: Dorfmueller, S. *et al.* Isolation of a recombinant antibody specific for a surface marker of the corneal endothelium by phage display. *Sci. Rep.* **6**, 21661; doi: 10.1038/srep21661 (2016).

## Supplementary Material

Supplementary Information
