# Supplementary material for: Isolation of a recombinant antibody specific for a surface marker of the corneal endothelium by phage display
Source: Sci Rep. 2016 Feb 23;6:21661. doi: 10.1038/srep21661 (PMC4763205; doi:10.1038/srep21661)
Supplement: Supplementary Information [file srep21661-s1.pdf]

Supplementary Information

Isolation of a recombinant antibody specific for a surface marker of the corneal endothelium by phage display

Simone Dorfmueller[1], Hwee Ching Tan[1], Zi Xian Ngoh[1], Kai Yee Toh[1], Gary Peh[2], Heng-Pei Ang[2], Xin-Yi Seah[2], Angela Chin[3], Andre Choo[3,4], Jodhbir Mehta[2], and *William Sun[5]

[1]Experimental Therapeutics Centre, Singapore. [2]Tissue Engineering and Stem Cell Group, Singapore Eye Research Institute. [3]Bioprocessing Technology Institute, Singapore. [4]Department of Biomedical Engineering, Faculty of Engineering, National University of Singapore. [5]Institute of Bioengineering and Nanotechnology, Singapore.

*Correspondence to wsun@ibn.a-star.edu.sg

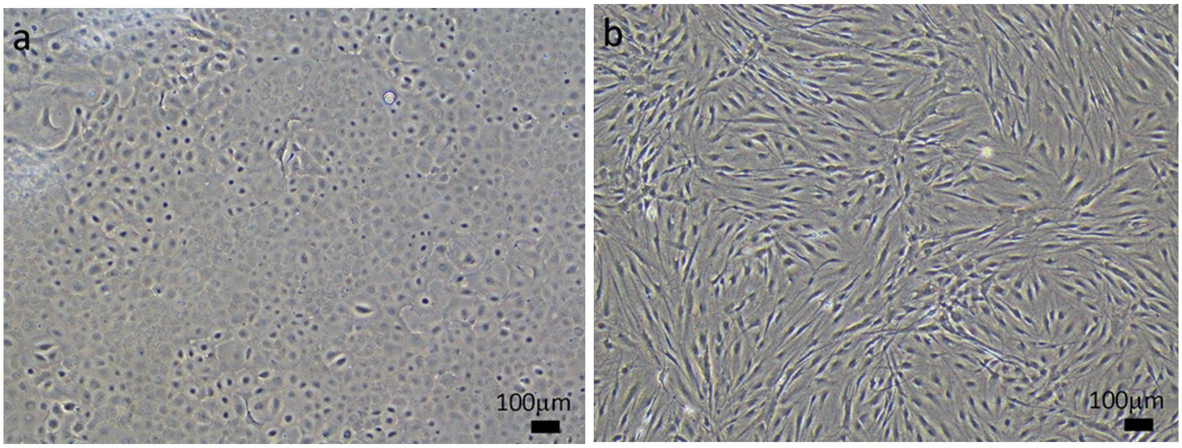

Supplementary Figure S1 Phase contrast images of cultured human corneal endothelial cells (**a**) and stromal fibroblasts (**b**).

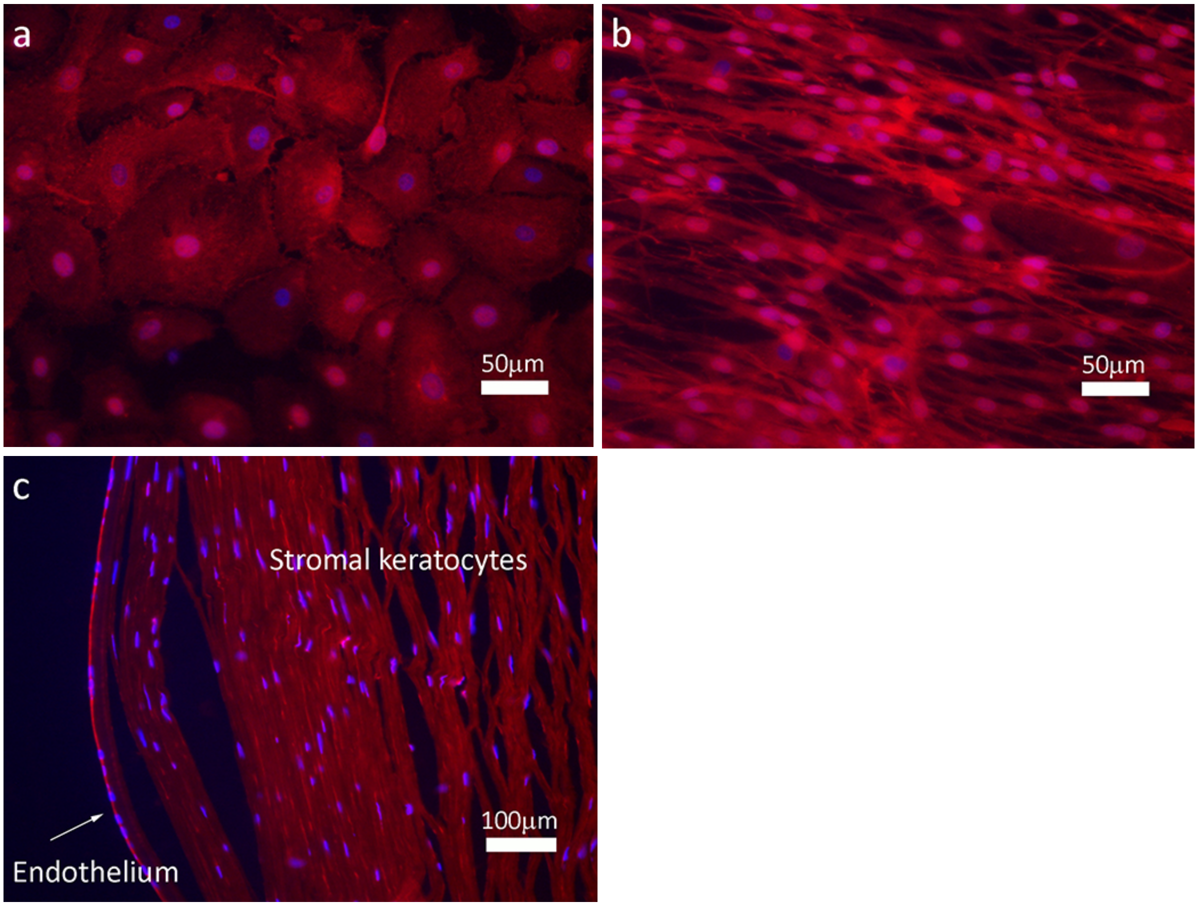

Supplementary Figure S2 Immunostaining of cultured hCEC (**a**), stromal fibroblasts (**b**), and human corneal section (**c**) using a non-specific scFV clone, C1M2-2-B2.

Supplementary Table S1: Amino acid sequences of the V_H CDRs for 20 positive clones from phage library screening

Clone	CDRH1	CDRH2	CDRH3
C3-1-A10	SYGIH	VISGSGGSTYYADSV	GGGVLDV
C3-2-D12	NYWIG	IIFPRDSETRFSPSF	LSGDMSGWPFDH
C2-1-H3	SNSAVWN	RTYYRSTWSNDYALSV	GVTAFDY
C2-3-H1	SNSAAWN	RTYYRSKWYNDYAVSV	GLGTGAFDI
C2-3-H2	SYAMH	AIGGGGTNTYYAGSV	GWLLFGD
C2-3-H6	SNSAAWN	RTYYRSKWYNDYAVSV	RGCTNGVCYFDY
C2-3-H7	NYYWS	YIYYSGSTNYNPSL	GVDMATINTFDI
C2-3-G9	NYAMN	GISGSGGTTYYADSV	GKGGGYNWRFDY
C2-3-H12	SNSAAWN	RTYYRSKWYNDYAVSV	GVTSFFDY
C2-4-D1	SYAMS	TIYYAGSNTYYADSV	GYYTFDY
C2-4-D4	SYAMH	AIGGGGTNTYYAGSV	GWLLFGD
C2-4-D9	RNGPVWN	RTYYRSKWYNDYAVSV	GQYSAFDI
C2-5-G4	SYAMS	LISGSGGSTYYADSV	GGGALGV
C1M2-2-B2	NARMN	LIKSKANGGATAYAAPV	ERAGAFHI
C1M2-2-B8	SYAMH	AIGGGGTNTYYAGSV	GWLLFGD
C1M2-2-H5	KFDMN	YIAATDNSVYYAESV	GHWDET
C1M2-2-B3	TFSSYAM	AIGGGGTNTYLAGSV	GWLLSGD
C1M2-1-D5	DSVSSNSAAW	RTYYRSKWYNDYAVSV	DHRPSHFDY
M3c-1-E7	TFTDDYAM	GISWNSGKIAYADSV	GPPLYSGHDSIHIPFDY
M3c-1-F11	TFTDDYAM	RINSDGSSTSNADSV	LVKMRAFDI

Supplementary Table S2: Amino acid sequences of the V_L CDRs for 20 positive clones from phage library screening

Clone	CDRL1	CDRL2	CDRL3
C3-1-A10	QGDSLRNYYAS	GNNNRPS	NSWDSSGNHVV
C3-2-D12	QGDSLGNSYAS	GINNRPS	NSRDSSGNHWV
C2-1-H3	SGNRSNIGSYYVS	TNDQRPS	AAWDDSLNGPVF
C2-3-H1	QGDSLRSYYAS	QDSKRPS	QAWDSSTFYV
C2-3-H2	TGSSGSIASTYVQ	EDNQRPS	QSYDTNNHGV
C2-3-H6	SGSSSNLGMNSVN	KSDQRPS	AAWDDSLNGYV
C2-3-H7	SGSSSNIGSNTVN	SDDQRPS	AAWDDSLNGYV
C2-3-G9	RASQSVYNYLA	DVSNRAT	QQRYEWPYT
C2-3-H12	TRSSGSIATYYVQ	EDNQRPS	QSYDSGNPPWV
C2-4-D1	QGDCLRGYYAS	GSNNRPS	NSRDNYSNHLV
C2-4-D4	TGSSGSIASTYVQ	EDNQRPS	QSYDLNNHVI
C2-4-D9	RSSQSLLHSDGYNYLD	LGSNRAS	MQALQTPKIT
C2-5-G4	RASRTISSYLN	ATFSLQS	QQAHSFPLT
C1M2-2-B2	SGSSSNIGSHNVY	KNNQRPS	AAWDNSVRGRV
C1M2-2-B8	SGSIASTYVQ	EDNQRPS	QSYDDGINWV
C1M2-2-H5	SGSIASNYVQ	DHNERPS	QSFDDNNQGV
C1M2-2-B3	TGSSGSITSTY	EDNQRPS	AAWDDSLSGYF
C1M2-1-D5	SGGSSDIGSNY	RNSQRPS	QSYDDGINWV
M3c-1-E7	SGSTSNIGKNY	DNNKRPS	GAWDTSLSAYV

M3c-1-F11	GGNNIGSKS	DDSDRPS	QVWDSSSDHLYV

Supplementary Table S3: Yields of scFV-AP fusion proteins after purification

**Clone**	**Yield (mg/L)**
C3-1-A10	0.08
C3-2-D12	0.34
C2-1-H3	0.20
C2-3-H1	0.19
C2-3-H2	Not inducible
C2-3-H6	0.37
C2-3-H7	0.18
C2-3-G9	0.12
C2-3-H12	0.19
C2-4-D1	0.08
C2-4-D4	Not inducible
C2-4-D9	0.12
C2-5-G4	0.08
C1M2-2-B2	0.39
C1M2-2-B8	0.20
C1M2-2-H5	0.0015
C1M2-2-B3	0.02
C1M2-1-D5	0.07
M3c-1-E7	0.03
M3c-1-F11	0.14

Supplementary Table S4: Sources of commercial RNAs for phage library construction.

**Company**	**Cat#**	**Item**
OriGene	HM1004	Human spleen mRNA, 5 µg
Biomed Diagnostics	636592	Clontech Human Blood, Peripheral Leukocytes Total RNA ,
Biomed Diagnostics	636580	Clontech Human Blood, Peripheral Leukocytes Total RNA
Biomed Diagnostics	636585	Clontech Human Fetal Spleen Total RNA
Biomed Diagnostics	636525	Clontech Human Spleen Total RNA
Invitrogen Singapore Pte Ltd	AM7970	Human spleen total RNA
Stratagene	540035	Human spleen total RNA
Stratagene	540021	Human lymph node total RNA
York-bio	N1101	Human total leukocytes total RNA
York-bio	N5141	Human adult spleen total RNA

Supplementary Methods

Isolation and culture of stromal fibroblasts

After the DM-endothelial layer has been peeled from the stroma, an 8.5 mm stroma button was obtained by trephination. The corneal epithelial layer was carefully scraped off using a scalpel blade. Stroma buttons were washed twice in a PBS-buffered antibiotic/antimycotic solution and enzymatically digested in collagenase overnight. The following day, stromal keratocytes released from within the stroma button were briefly washed twice with PBS, seeded onto cell culture flask coated with FNC coating mixture, and cultured in F99 medium. The exposure to a serum-supplemented medium transformed the corneal stromal keratocytes into fibroblasts. Culture medium was refreshed every two days, and confluent fibroblast cultures were passaged using TrypLE Express (Life Technologies) in a 1:5 split ratio. Stromal fibroblasts from several donor corneas were pooled for this study.

Cell based scFv and phageELISA

To monitor efficient subtraction of fibroblast specific and enrichment of hCEC specific phages, polyclonal phage libraries before and after subtraction and/or panning round were collected and tested on fibroblasts and hCEC. Cells were grown to 90% confluency in FnC-coated 96 well plates (6K per well for hCEC, 4K per well for stromal fibroblasts). Cells were fixed with 10% formaldehyde for 10min, blocked with 400 μl 3% BSA per well for 2h at RT and washed 3 times with PBS-Tween20 (0.05%) for 5min. Polyclonal phages were added in PBS and incubated at RT for 2h. KM13 helper phage was included as negative control. After washing 3 times with PBS-T for 5min, bound phages were detected by HRP conjugated anti-M13 antibody (1:5000 in 2% BSA; GE Healthcare) and the assay was developed by addition of 100 μl TMB (Promega). Reaction was stopped by the addition of 100 μl H_2SO_4 and reading taken at OD_{450nm}.

To screen for hCEC-specific monoclonal scFv, well-isolated colonies were inoculated o/n at 37°C in 2xTY, ampicillin, 1% glucose. A vector clone was used as negative control. The overnight cultures were mixed 1:6 with 2xTY, ampicillin, 1% glucose and incubated for 4-5h at 37°C. Medium was exchanged with 2xTY, ampicillin, 0.4 M sucrose, 1 mM IPTG, cultures were incubated o/n at 30°C and pelleted. 100 μl of the supernatant was tested in parallel on cultivated hCEC and fibroblast as described for the phage ELISA, except that the detection was done by a HRP conjugated c-myc specific antibody (1:10 000 in PBS: Pierce).

Immunohistochemistry

For immunohistochemistry of frozen sections, human donor cornea was rinsed in PBS twice and immersed in OCT medium and frozen at -80°C until sectioning. Eight-micron thick sections were cut using a MicromHM550 cryostat and collected on glass slides and air-dried before storage at -80°C. For in vitro staining of primary hCECs and corneal stromal fibroblasts, cells were seeded (4k/well for hCEC and stromal fibroblasts) on Multi-test slides (MP Biomedicals), pre-coated with FnC. Cells or tissue sections were fixed in 10% formaldehyde, washed 3 times 5 min with PBS, blocked with 10% goat serum in PBS (Millipore) for 1h at RT and washed 3 times with PBS. Slides were dried and wells and tissue were surrounded with a PAP pen. After 1h at RT incubation with primary antibodies (scFv AP or IgG) in 4% Goat serum probes were washed 3 times with PBS and incubated 15min with DAPI (1:10 000 in PBS; Invitrogen). After 3 times washing with PBS and 1 time with PBS-Tween20 (0.05%), bound scFv-AP was detected by the Vector Red alkaline phosphatase substrate kit (Vector Labs), and bound IgG by a goat anti-human Alexa488 conjugated secondary antibody (1:750; Invitrogen). After adding 3ul of ProLong Gold (Invitrogen), slides were covered with a cover slip and viewed under a fluorescence microscope.

Flow cytometry

Primary hCECs and fibroblasts were dissociated as described earlier by, blocked with 10% goat serum in PBS at 37°C for 15min. Cells were washed 1 time with PBS and incubated for 45 min at 4°C with 4 μg/ml scFv or IgG conjugated to DyLight™ 488 (Innova Bioscience). Conjugation of fluorescent labels was performed according to manufacturer's instructions. Cells were washed 2 times with PBS, resuspended in 0.2 ml PBS and analyzed by flow cytometry (Accuri C6, BD Bioscience). Labelled hCECs and fibroblasts were analyzed separately and gating was performed to exclude debris and doublets.

In-gel tryptic digest and mass spectrometry

The excised protein band was pre-incubated with 150 µl of washing solution containing 2.5mM ammonium bicarbonate and 50% acetonitrile (ACN) for at least 24 h at 4oC. Fresh washing solution was replaced and the gel band was incubated at room temperature for 10 min before subjecting it to reduction in buffer containing 10mM dithiothreitol DTT)/100mM ammonium bicarbonate for 1 h at 56oC. The reducing solution was aspirated from the well, followed by the addition of 55mM iodoacetic acid (IAA)/100mM ammonium bicarbonate for a period of 45 min at room temperature in order for alkylation to occur. The gel was washed with 100µl of 100mM ammonium bicarbonate followed by ACN at intervals of 10 min. The washing process was repeated and the gel was finally dried by Speedvac (Savant). Trypsin solution (10µl of 20nl/ml) was added to each well and left overnight at 37 oC for 16 h. The peptides were extracted with 50% acetonitrile and 5% formic acid and sonicated on the water-bath sonicator for 10 min. After drying down the sample using Speedvac , the pellet was reconstituted in 5.5 µl of 2% methanol, 1% formic acid for LCMS/MS (Thermos LTQ Orbitrap Elite Mass Spectrometry). The results were searched against the human database on Mascot.
